# Thank you to the *Journal of the Medical Library Association* reviewers in 2020

**DOI:** 10.5195/jmla.2021.1200

**Published:** 2021-04-01

**Authors:** Katherine G. Akers

**Affiliations:** 1 JMLA@journals.pitt.edu, Editor-in-Chief, *Journal of the Medical Library Association*

## Abstract

The *Journal of the Medical Library Association (JMLA)* sincerely thanks the 214 peer reviewers in 2020 who helped vet and improve the quality of work published in our journal.

The *Journal of the Medical Library Association (JMLA)* sincerely thanks the 214 peer reviewers in 2020 who helped vet and improve the quality of work published in our journal.

*JMLA* is always looking to expand our pool of reviewers with expertise in specific domains in health sciences librarianship research and practice. If you would like to serve as a peer reviewer for *JMLA,* please indicate your interest to an assistant editor, an associate editor, or the editor-in-chief.

## 2020 *JOURNAL OF THE MEDICAL LIBRARY ASSOCIATION* REVIEWERS

Karen Sue Alcorn

Kristine M. Alpi, AHIP

Kate Anderson

Katelyn Angell

Marilia Y. Antunez, AHIP

Nell Aronoff

Marie T. Ascher

Erinn Aspinall, AHIP

Paul Bain

Caitlin J. Bakker, AHIP

Jill Barr-Walker

Joan Bartlett

Robert D. Beckett

Cynthia Beeler, AHIP

Skye Bickett, AHIP

Paul Blobaum

Roxanne Bogucka, AHIP

Sarah Bonato

Jill Boruff, AHIP

Alexandria Leigh Brackett, AHIP

Wichor Bramer

Marci Brandenburg

Ellen Brassil, AHIP

Stewart Brower, AHIP

Heather L. Brown

Roy Eugene Brown, AHIP

Krystal Bullers, AHIP

Christopher Sean Burns

Sarah Cantrell, AHIP

Nicole Capdarest-Arest, AHIP

Sarah L. Carnes, AHIP

Alexander J. Carroll, AHIP

Tallie Casucci

Susan Cavanaugh

Thane Chambers

Robin E. Champieux

Liza Chan

Amy Chatfield

Marina Chilov

Heather Coates

Jamie L. Conklin

Marisa Conte, AHIP

John W. Cyrus

Nicole Dalmer

Prudence W. Dalrymple, AHIP, FMLA

Jennifer Davis

Ariel Deardorff

Sandy De Groote, AHIP

Basia Delawska-Elliott, AHIP

Robin Desmeules

Kerry Dhakal

Tara Douglas-Williams, AHIP

Chris Eaker

Martha Earl, AHIP

Erin R.B. Eldermire

Keith D. Engwall, AHIP

Helen-Ann Brown Epstein, AHIP, FMLA

Nina Exner

Kelly Farrah, AHIP

Lisa Federer, AHIP

Michelle Fiander

David Flynn

Susan A. Fowler

Marian Fragola

Jacqueline Freeman

Melissa Christine

Funaro Julie

May Glanville

Emily J. Glenn

Sally Gore

Kelsey Grabeel, AHIP

Adelia Grabowsky

Vera Granikov

Alyssa Grimshaw

Jean Gudenas, AHIP

Karen Elizabeth

Gutzman Rosie Hanneke, AHIP

Gale G. Hannigan, AHIP

Conor Hanrahan

Ardis Hanson, AHIP

Eli Harriss

R. Brian Haynes

Emma Heet

Melissa Helwig

Margaret Henderson, AHIP

Heather N. Holmes, AHIP

Kristi L. Holmes

Allison M. Howard, AHIP

Carmen Howard

Carrie L. Iwema, AHIP

Rebecca Jacob

Sarah T. Jewell

Phill Jo, AHIP

Timothy Johnson

Emily M. Johnson-Barlow, AHIP

Emily Paige Jones

Kellie N. Kaneshiro, AHIP

Samantha J. Kaplan

Jill R. Kavanaugh, AHIP

Sujin Kim

Elizabeth J. Kiscaden, AHIP

Michele Klein-Fedyshin, AHIP, FMLA

Mary Lou Klem

Helge Knüttel

Rachel Amelia

Santose Koenig

Iris Kovar-Gough, AHIP

Chana Kraus-Friedberg, AHIP

Jennifer Langford

Mariana Lapidus

Stefanie Lapka

Janna C. Lawrence, AHIP

Brenda M. Linares, AHIP

Anne M. Linton, AHIP

Elizabeth R. Lorbeer, AHIP

Diane L. Lorenzetti

Ya-Ling Lu

Irene (Rena) Machowa Lubker, AHIP

Brady Daniel Lund

Mark MacEachern

Keith C. Mages, AHIP

Tara R. Malone

Heather J. Martin, AHIP

Lisa Mastin

Sarah McClung

Karen McElfresh, AHIP

Bethany S. McGowan, AHIP

Melanie McGurr

Dina J. McKelvy, AHIP

Caitlin Meyer

Misa Mi, AHIP

Jolene M. Miller, AHIP

Christian I.J. Minter

Heather K. Moberly, AHIP

Nisha Mody

Elizabeth Moreton

Anna Beth Morgan, AHIP

Rebecca Anne

Morin Martin Morris

Joanne Marie Muellenbach, AHIP

Kavita S. Mundle

Bethany Myers, AHIP

Amanda Nevius

Sawyer Newman

Joey Nicholson

Annie Nickum, AHIP

Melanie J. Norton

Kelly K. O’Brien

Robin O’Hanlon

Eugenia Opuda

Ani Orchanian-Cheff

Rob O’Reilly

Peace Ossom Williamson, AHIP

Janet Papadakos

Robin M.N. Parker

Pete Pascuzzi

Emily Patridge, AHIP

Gerald J. Perry, AHIP, FMLA

Jonna Peterson

JJ Pionke

T. Scott Plutchak, AHIP, FMLA

Kimberly R. Powell

Zahra Premji

Alexandria Quesenberry

Rebecca Raszewski, AHIP

Kevin Read

Robyn B. Reed, AHIP

Melissa L. Rethlefsen, AHIP

Rebecca Reznik-Zellen

Joshua Richardson

Morwenna Rogers

Amanda Ross-White, AHIP

Stephanie Clare Roth, AHIP

Sarah Safranek

José Antonio Salvador-Oliván

Alexandra Sarkozy

Cathy Sarli, AHIP

Cynthia Marie Schmidt

Jan W. Schoones

Stephanie J. Schulte

Amanda Scull

Claire Sharifi

Tracy C. Shields, AHIP

Mary Simons

Erin M. Smith

Judy Spak

Edwin Vincent Sperr Jr., AHIP

Stuart Spore

R. Grant Steen

Elizabeth Stellrecht

Judy Carol Stribling, AHIP

Julia Stumpff

Yalin Sun

Alisa Surkis

Anthea Sutton

Stephanie M. Swanberg, AHIP

Natalie Tagge

Maria Tan

Nicole Theis-Mahon, AHIP

Holly Thompson

Stephanie Tomlinson, AHIP

Elizabeth Kay Tompkins

Lauren Topper

Angela Faye Tucker, AHIP

Ana Ugaz

Christine Urquhart

Saeideh Valizadeh-Haghi

Emily Vardell, AHIP

Megan von Isenburg, AHIP

Stacey E. Wahl

Jennifer Watling Neal

Monique Wessels

Jeff D. Williams, AHIP

Christina L. Wissinger

Andrea Wright

Joe Wu

